# Revisiting the Death and Autopsy of King George II

**DOI:** 10.1055/s-0041-1729915

**Published:** 2021-12-08

**Authors:** Yota Suzuki, Abe DeAnda

**Affiliations:** 1 Department of Surgery, University of Texas Medical Branch Galveston, Galveston, Texas; 2 Division of Cardiovascular and Thoracic Surgery, University of Texas Medical Branch Galveston, Galveston, Texas

**Keywords:** aortic dissection, coronary artery disease, ventricular rupture

## Abstract

It is commonly accepted that King George II died of an acute aortic dissection. The origin of this association derives from retelling of the official autopsy performed by Dr. Frank Nicholls. While there is no doubt that King George II did have a Stanford Type A dissection, critical descriptions in the report point to a more likely cause of death.

## Introduction

Aortic surgery, as in other medical specialties, has a lore and tradition that include the apocryphal. While Osler's quote “There is no disease more conducive to clinical humility than aneurysms of the aorta” is well documented 1 and often included in treatises on aortic disease, perhaps the second most common reference is the notation that King George II died of an acute aortic dissection. The origin of this association derives from the official autopsy performed by Dr. Frank Nicholls, 2 and while Dr. Nicholls describes the postmortem findings in detail, the attribution of the cause of death may have been incorrect. This brief paper revisits the autopsy with reference to the original report published by Dr. Nicholls in the proceedings of the Royal Society to elaborate on the more likely cause of death.

### Frank Nicholls

Frank Nicholls, MD, FRS, was an English physician, born in 1699. He attended Exeter College at Oxford, obtaining his Bachelor of Arts degree in classics and physics at the age of 19 years, he got his Master's degree at 21 years, a Bachelors of Medicine at 25 years, and his Doctorate at 30 years. Prior to graduating in medicine, he lectured at Oxford in anatomy, mostly devoted to what was then referred to as minute anatomy and demonstrated the structure of small blood vessels. In 1727, he presented to the Royal Society, a paper and demonstration regarding the formation of aneurysms and consequences, including frank rupture and “rupture of the internal coats of the artery,” probably one of the earliest descriptions of an aortic dissection albeit experimentally contrived. 3 In this demonstration to the Royal Society, he pressurized a cadaveric pulmonary artery and showed that while the “external coating” (i.e., the adventitial layer) was intact despite the aneurysmal distention, the “internal coating” (presumably the intima and media) tore. His academic career continued with distinction, and in 1753, he was appointed as physician to King George II. This appointment was probably not happenstance, as his father-in-law, Richard Mead, had previously filled the position of physician to the King.

### The Death and Autopsy of King George II

As has been described numerous times, on the morning of October 25, 1760, the King arose from bed, had a cup of hot chocolate, and went alone to his privy for a bowel movement. His valet heard a loud crash and found him unresponsive on the floor, his only sign of trauma being a cut on his face presumably related to his fall. The house surgeon, Mr. Andrews, was immediately brought in and as might be expected, was unable to revive the King despite multiple attempts at bloodletting. As it was apparent that the King had died, and as was the custom, an autopsy was ordered to rule out any nefarious cause of death. To this end, Dr. Nicholls was directed to perform the autopsy and embalming the following day.

**Fig. 1:**
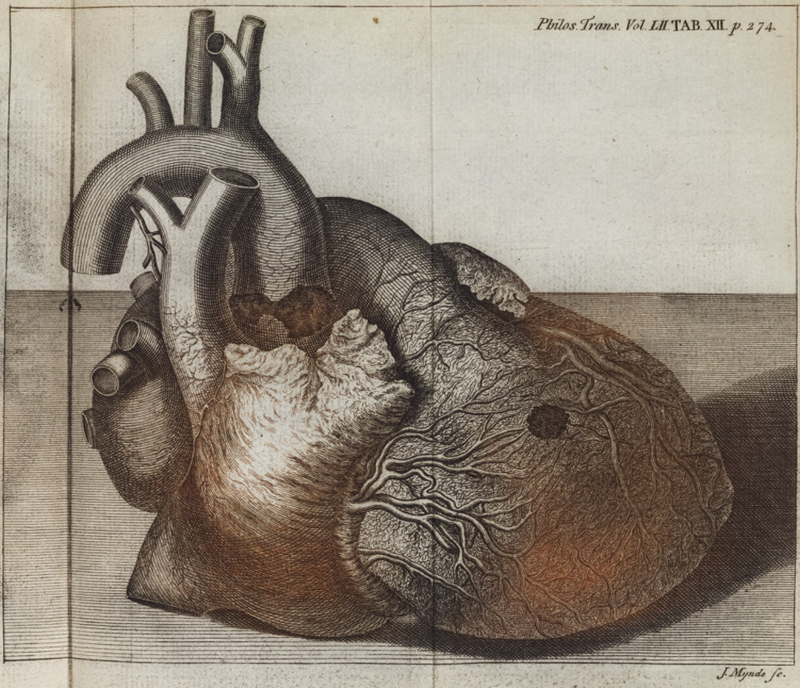
Two-toned engraving (by J. Mynde) which accompanied Dr. Nicholls' description of the autopsy findings of King George II.

The abdomen was opened first, with no abnormalities noted with the exception “… some hydatides (or watery bladders) were found between the substance of each kidney, and its internal coat.” Upon opening the chest, hemopericardium was discovered, the blood coagulated, “… nearly sufficient to fill a pint cup … upon removing this blood, a round orifice appeared in the middle of the upper side of the right ventricle of the heart, large enough to admit the extremity of the little finger” ( Fig. 1 ). Dr. Nicholls also noticed that while intact, the aorta, pulmonary artery, and right ventricle were “… stretched beyond their natural state.” Dr. Nicholls rightly surmised that the proximal cause of death was cardiac tamponade and that the King “… must, therefore, have dropped down, and died instantaneously.” Upon opening the aorta, an approximately 4-cm long “transverse fissure on its inner side” was identified, “through which some blood had recently passed.”

As opposed to the lore that King George II died of aortic dissection that ruptured into the pericardial sac, 4 Dr. Nicholls clearly described right ventricular rupture with aortic dissection “… as the immediate cause of … bursting.” Importantly, Dr. Nicholls did not note the aorta to have ruptured. As a natural question, Dr. Nicholls was perplexed with the association of the dissection and ventricular rupture and proposed a hypothesis in the same manuscript. From his previous observations on the nature of aneurysms, Dr. Nicholls theorized that the root aneurysm seen was long standing, and the superimposed acute dissection caused “… a more extraordinary and violent distention, immediately antecedent to the bursting of the ventricle.” It was this “violent distention” that led the aorta to compress the pulmonary artery resulting in “… an increased opposition to the passage of the blood out of the right ventricle” which subsequently resulted in a sudden increase in right ventricular pressure and consequent rupture.

### Evidence against the Dissection as the Immediate Cause of Death

The possibility that King George II did not die from an acute aortic dissection is suggested by the following considerations. First, as noted in the description of the autopsy, the aorta did not rupture, the right ventricle ruptured. Surgeons have long observed bleeding from the epicardial surface of either ventricle associated with an acute aortic dissection, typically originating from tracking of subadventitial blood from the aorta onto the surface of the heart, not as the result of a hole in the ventricle. Second, contrary to Dr. Nicholls' hypothesis, with modern experience with pulmonary hypertension (and with real-time monitoring with either right-heart catheterization or continuous monitoring with a pulmonary artery catheter), we know that systemic pulmonary artery pressure or a pressure surge in the artery will not result in rupture or perforation of the right ventricle. Instead, such high pulmonary artery pressures result in acute failure of the ventricle, for example, the pathophysiology seen with a large pulmonary embolism. Third, King George II had been having cardiac symptoms for some time with Dr. Nicholls noting in his report that “… his Majesty had, for some years, complained of frequent distresses and sinkings about the region of the heart … his pulse was, of late years, observed to fall very much upon bleeding.”

While acknowledging that the King did have a Stanford Type A aortic dissection, from the autopsy report, the possibility arises that his demise was due to coronary artery disease. Angina would not be described by Heberden 5 until 1768 (and published in 1772) and the relationship of angina to coronary artery disease would have to wait another 140 years. 6 Thus, this etiology was not available to Dr. Nicholls to aid in his diagnosis. Postinfarction myocardial rupture is well described albeit less common in the present era due to early intervention and is even less common on the right side of the heart. The presence and etiology of coronary artery disease in King George II is unknown and may have been secondary to atherosclerotic disease or may have been due to the chronic dissection itself. While acute ischemia secondary to aortic dissection is typically due to occlusion or disruption of the ostia of the coronaries, dissection of one or both coronaries can lead to chronic ischemia. One bit of evidence to support the coronary artery disease hypothesis is the illustration by Dr. Nicholls ( Fig. 1 ) which suggests the perforation to have occurred in a watershed zone on the free wall of the right ventricle.

As an aside, there are two other historical notations in the autopsy report. First, the “hydatides” as noted by Dr. Nicholls, while at the time just a curiosity, is the earliest description of an association between renal cystic disease and aortic disease. This entity was recently described by Brownstein et al 7 with the authors noting the prevalence of renal cysts seen in patients undergoing surveillance scans for aneurysmal disease, compared with the general population. Second, the accompanying illustrations to Dr. Nicholls' paper were printed in two colors (brown and sanguine) and are probably the first color-printed plates in a major scientific periodical.

## Conclusion

We appreciate Dr. Nicholls' precise and detailed description of the autopsy that allows us to make a scientific reconsideration of the cause of death of King George II, using our current understanding of pathophysiology which evolved over the past 260 years since this momentous event.

## Notes

Editor's Commentary
*Jorge Mascaro, MD*

*Department of Cardiothoracic Surgery, Queen Elizabeth University Hospital NHS Foundation Trust, Birmingham, United Kingdom*
I would like to congratulate our colleagues for this historical revision. Although the real causes for the loss of King George II remain unclear, the analysis provided helps to better understand, with the application of current knowledge, events that had been explained with only limited information available. It also reminds us of the brilliant minds of the past; with very limited information they could arrive to quite precise diagnosis. What we are today we owe to the many scientists of the past.
